# Building a Machine Learning Model to Predict Postpartum Depression from Electronic Health Records in a Tertiary Care Setting

**DOI:** 10.3390/jcm14186644

**Published:** 2025-09-20

**Authors:** Zhitu Ma, Michael Horvath, David Michael Stamilio, Kobby Sekyere, Metin Nafi Gurcan

**Affiliations:** 1 Clinical and Translational Science Institute, Wake Forest University School of Medicine, Winston-Salem, NC 27101, USA; zhma@wakehealth.edu (Z.M.); mhorvath@wakehealth.edu (M.H.); ksekyere@wakehealth.edu (K.S.); 2 Department of Obstetrics and Gynecology, Section of Maternal-Fetal Medicine, Wake Forest University School of Medicine, Winston-Salem, NC 27101, USA; dstamili@wakehealth.edu; 3 Center for Artificial Intelligence Research, Wake Forest University School of Medicine, Winston-Salem, NC 27101, USA

**Keywords:** depression, postpartum, machine learning, electronic health record, health inequalities

## Abstract

**Background**: Postpartum depression is a common mental health condition that can occur up to one year after childbirth. Recent studies have increasingly used machine learning techniques to predict its occurrence; however, few have comprehensively explored the use of electronic health record data, particularly in tertiary care settings where such data can be fragmented. **Methods**: We analyzed electronic health record data from 12,284 women who delivered at The Birth Center at Atrium Health Wake Forest Baptist Medical Center, excluding those with missing data or no prenatal or postpartum visits. To define the target variable, we examined different combinations of depression screening tools (Edinburgh Postnatal Depression Scale and Patient Health Questionnaire-9), along with diagnosis codes specific to postpartum depression. We then trained a random forest classification model to predict postpartum depression. **Results**: The model achieved an area under the receiver operating characteristic curve of 0.733 ± 0.008, which is comparable to previous studies. Adding socioeconomic features from census tract data did not improve predictive performance, underscoring the importance of individual-level data. Incorporating national survey data, such as the Pregnancy Risk Assessment Monitoring System, also did not improve performance due to limited overlap in data features. Interestingly, model performance was slightly lower among Hispanic patients (area under the curve = 0.713 ± 0.040), although this difference was not statistically significant (*p* = 0.17), likely due to the small sample size. A similar, but statistically significant trend was observed in the larger national survey dataset (area under the curve = 0.699 ± 0.019 for Hispanic patients versus 0.735 ± 0.010 for White patients, *p* < 0.01). **Conclusions**: While our model demonstrates moderate predictive capability, further validation and prospective testing are needed before clinical implementation. This work also identified an optimal approach for digital phenotyping postpartum depression in electronic health record data and highlighted key gaps in data quality and completeness. These findings emphasize the importance of robust data when developing predictive models for real-world clinical use.

## 1. Introduction

Postpartum depression (PPD) is a prevalent complication affecting mothers after childbirth, with an estimated prevalence of 17.22% globally and 18.56% in the United States [1]. Given that many cases may remain undiagnosed, the true prevalence rate could be even higher [2,3]. Postpartum depression not only affects maternal health shortly after birth [4], but also impacts parent–child relationships and is associated with negative outcomes for children in the long term [5,6]. A wide range of risk factors have been identified, including past depression, stressful life events, marital dissatisfaction, inadequate social support, mode of delivery, socioeconomic status, obesity, and others [7,8,9,10]. These findings underscore the multifactorial nature of postpartum depression and the need for reliable early identification approaches.

In recent years, there has been a rapid increase in the application of machine learning (ML) techniques in healthcare [11]. Several models have been developed to predict and understand PPD (see [12] for a comprehensive review). While many of these studies are based on publicly available datasets [13,14] or prospective cohort studies [15,16], constructing cohorts directly from electronic health records (EHR) remains an attractive approach that could allow researchers to work with larger datasets of real-world patient populations more efficiently [17]. However, several challenges remain unaddressed, including the lack of clear comparisons between different methods for phenotyping, limited availability of granular socioeconomic information, insufficient cross-cohort comparisons, and the limited availability of data for certain patient subgroups.

This study addresses these gaps by developing and evaluating a machine learning model for predicting postpartum depression using electronic health record data from a large academic tertiary care birth center. Specifically, we examined strategies for defining the target variable from patients’ health records, assessed the value of incorporating census tract-level socio-economic data, and tested model generalizability using national survey data. We also evaluated predictive performance across racial and ethnic groups to explore potential disparities in model predictability.

The objective of this work is to advance the understanding of how electronic health records-based models can be used to identify mothers at risk for postpartum depression, and to provide empirical insights into the opportunities and limitations of these approaches for future clinical applications.

## 2. Materials and Methods

To operationalize this work, we first constructed a well-defined patient cohort from our institutional EHR system, which is implemented in Epic, using the PCORnet Common Data Model (Section 2.1 and Section 2.2). We then developed a Random Forest classifier based on demographic, clinical, and diagnostic features extracted from structured EHR data, with model evaluation performed using repeated cross-validation and AUC as the primary metric (Section 2.3.2, Section 2.3.3 and Section 2.3.4). Additional features were derived from linked census tract data and the publicly available PRAMS dataset to explore their potential additive value (Section 2.4 and Section 2.5). Details of model performance, feature importance, and subgroup analyses are provided in Section 3. For reproducing and sharing purposes, all of our computer codes used for data preprocessing, modeling, and evaluation will be available at https://github.com/wake-forest-ctsi/ammi (accessed on 18 September 2025).

### 2.1. Cohort Selection

**Figure 1:**
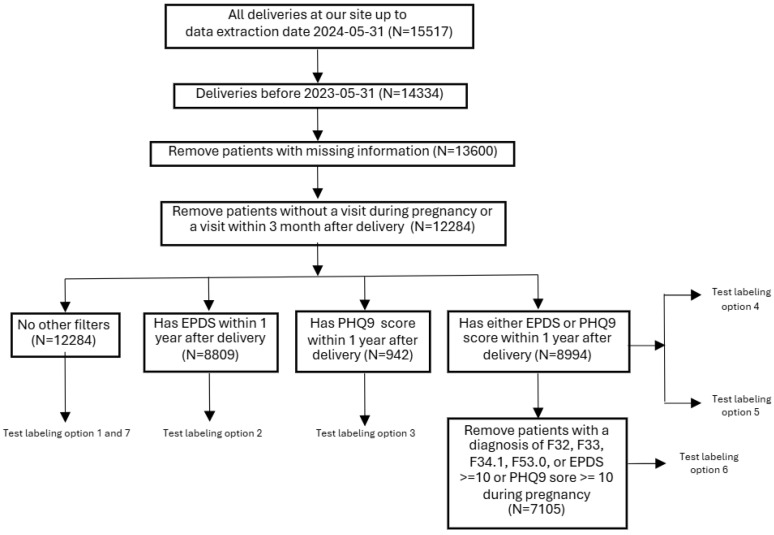
Cohort Selection and Data Preprocessing Overview. The cohort was defined through the following steps: (1) Restricting the cohort to women whose delivery occurred on or before 31 May 2023; (2) Excluding patients with missing data; and (3) Including only those women who had at least one visit during pregnancy and one visit within three months postpartum. Further filtering was applied to align with the specific definition of the target variable. For additional details, refer to Table 2 and Section 2.3.1.

Figure 1 shows the overall process of cohort selection and data preprocessing. We collected EHR data from all women who delivered at The Birth Center at Atrium Health Wake Forest Baptist Medical Center from its inception in 2019 through 31 May 2024 (*n* = 15,517). Our primary focus is predicting postpartum depression within one year after delivery; therefore, we restricted the cohort to women whose delivery occurred on or before 31 May 2023 (*n* = 14,334). We then excluded patients with missing data for key candidate predictive variables listed in Table 1 (*n* = 13,600), specifically those without any records of BMI, weight, height, smoking status, or tobacco use during the feature selection period (from two years prior to delivery up to the time of delivery). For parity and PHQ-9/EPDS scores, missing values were imputed as 0, and additional indicator variables were created to flag imputed cases. All other variables were complete. Following [18], we further restricted the dataset to include only women who had at least one prenatal visit and one visit within three months postpartum within the health system (*n* = 12,284). This filtering step ensures that the cohort consists of patients more likely to have established healthcare within our health system.

**Table 1:** (**a**) Characteristics of the cohort for continuous predictive variables. Postpartum depression (PPD) is defined according to Method 5 in Table 2. Average values and standard deviations are reported, except for parity, for which the median and 25th/75th percentiles are listed. All features were calculated from two years prior to delivery up to the time of delivery. (**b**) Same as Table 1a, but for categorical variables. All features were calculated from two years prior to delivery up to the time of delivery. The unadjusted odds ratios (OR) of PPD with 95% confidence intervals (CI) are listed.

(a)			
Variable	PPD (N = 1986; 22.1%)	Non-PPD (N = 7008; 77.9%)	*p*-Value
Mother age	28.2 ± 5.9	28.9 ± 5.9	*p* < 0.001
Gestation age at delivery (in days)	268.0 ± 13.0	269.5 ± 10.2	*p* < 0.001
Parity	1 (0–2)	1 (0–2)	*p* = 0.002
BMI	32.1 ± 8.0	31.5 ± 12.4	*p* = 0.005
Mother height	64.2 ± 2.8	63.9 ± 2.8	*p* < 0.001
Prenatal care visit			
Counts of visits	36.6 ± 16.3	32.5 ± 14.3	*p* < 0.001
Counts of Ambulatory Visit (AV)	17.8 ± 7.8	16.3 ± 6.9	*p* < 0.001
Counts of Inpatient Hospital Stay (IP)	0.7 ± 0.7	0.6 ± 0.7	*p* < 0.001
Counts of Emergency Department (ED) visits	0.6 ± 1.3	0.4 ± 0.9	*p* < 0.001
Counts of Telehealth (TH) visits	0.2 ± 0.9	0.1 ± 0.6	*p* < 0.001
Counts of Other (OT) visits	17.3 ± 8.6	15.1 ± 7.8	*p* < 0.001
PHQ * score max			
phq_21012948	0.4 ± 0.9	0.1 ± 0.5	*p* < 0.001
phq_21012949	0.5 ± 0.9	0.1 ± 0.5	*p* < 0.001
phq_21012950	1.8 ± 1.2	1.3 ± 1.2	*p* < 0.001
phq_21012951	2.0 ± 1.1	1.5 ± 1.2	*p* < 0.001
phq_21012953	1.5 ± 1.2	1.0 ± 1.2	*p* < 0.001
phq_21012954	1.4 ± 1.2	0.9 ± 1.1	*p* < 0.001
phq_21012955	1.3 ± 1.2	0.9 ± 1.1	*p* < 0.001
phq_21012956	0.9 ± 1.1	0.6 ± 1.0	*p* < 0.001
phq_21012958	0.3 ± 0.7	0.2 ± 0.6	*p* = 0.041
phq_21012959	11.9 ± 6.8	8.6 ± 6.7	*p* < 0.001
Edinburgh Postnatal Depression Screen total score max **			
EPDS_99046_max	7.9 ± 7.0	3.7 ± 4.4	*p* < 0.001
EPDS_71354_max	5.1 ± 5.8	1.9 ± 3.1	*p* < 0.001
**(b)**			
**Variable**	**PPD** **(N = 1986; 22.1%)**	**Non-PPD** **(N = 7008; 77.9%)**	**OR (CI)**
Mother self-reported race/ethnicity			
Black	463 (23.3)	1497 (21.4)	1.12 (0.99, 1.26)
Hispanic	196 (9.9)	1287 (18.4)	0.49 (0.42, 0.57)
White	1153 (58.1)	3447 (49.2)	1.43 (1.29, 1.58)
Other/Unknown	190 (9.6)	891 (12.7)	0.73 (0.62, 0.86)
Insurance type			
Medicaid	971 (48.9)	3340 (47.7)	1.05 (0.95, 1.16)
Managed Care (Private)	1075 (54.1)	3627 (51.8)	1.10 (1.00, 1.22)
Self-Pay	972 (48.9)	3718 (53.1)	0.85 (0.77, 0.94)
Legal Liability/Liability Insurance	13 (0.7)	54 (0.8)	0.85 (0.46, 1.56)
Medicare	21 (1.1)	38 (0.5)	1.96 (1.15, 3.35)
Private health insurance—other commercial Indemnity	41 (2.1)	142 (2.0)	1.02 (0.72, 1.45)
TRICARE (CHAMPUS)	18 (0.9)	64 (0.9)	0.99 (0.59, 1.68)
Other Government (Federal, State, Local not specified)	26 (1.3)	96 (1.4)	0.96 (0.62, 1.48)
Worker’s Compensation	6 (0.3)	19 (0.3)	1.11 (0.44, 2.79)
Medicaid Applicant	17 (0.9)	123 (1.8)	0.48 (0.29, 0.80)
Delivery mode			
Vaginal, Spontaneous	1086 (54.7)	4140 (59.1)	0.84 (0.76, 0.92)
C-Section, Low Transverse	764 (38.5)	2413 (34.4)	1.19 (1.07, 1.32)
Vaginal, Vacuum (Extractor)	31 (1.6)	145 (2.1)	0.75 (0.51, 1.11)
VBAC, Spontaneous	25 (1.3)	105 (1.5)	0.84 (0.54, 1.30)
Vaginal, Forceps	27 (1.4)	65 (0.9)	1.47 (0.94, 2.31)
C-Section, Classical	16 (0.8)	26 (0.4)	2.18 (1.17, 4.07)
C-Section, Low Vertical	10 (0.5)	31 (0.4)	1.14 (0.56, 2.33)
Vaginal, Breech	14 (0.7)	23 (0.3)	2.16 (1.11, 4.20)
C-Section, Other Specified Type	4 (0.2)	21 (0.3)	0.67 (0.23, 1.96)
C-Section, Unspecified	3 (0.2)	13 (0.2)	0.81 (0.23, 2.86)
Smoking status			
smoking	772 (38.9)	2036 (29.1)	1.55 (1.40, 1.72)
tobacco	16 (0.8)	48 (0.7)	1.18 (0.67, 2.08)
Obesity from diagnosis code			
morbid (E66.01)	374 (18.8)	1006 (14.4)	1.38 (1.21, 1.58)
obese (E66.09, E66.8, E66.9)	610 (30.7)	1904 (27.2)	1.19 (1.07, 1.33)
overweight (E66.3)	27 (1.4)	76 (1.1)	1.26 (0.81, 1.96)
Obesity complicating pregnancy, childbirth, and the puerperium (O99.21)	843 (42.4)	2665 (38.0)	1.20 (1.09, 1.33)
Delivery year			
2018–2019	357 (18.0)	1159 (16.5)	1.11 (0.97, 1.26)
2020	489 (24.6)	1619 (23.1)	1.09 (0.97, 1.22)
2021	654 (32.9)	2314 (33.0)	1.00 (0.90, 1.11)
2022	344 (17.3)	1326 (18.9)	0.90 (0.79, 1.02)
2023	142 (7.2)	590 (8.4)	0.84 (0.69, 1.01)

* These variables include scores for each question in the PHQ-9 and the total score. Questions in the PHQ are listed in Table S2. ** Two EPDS total scores are listed in our EHR with different entry names: Edinburgh Postnatal Depression Scale Total (71354) and Edinburgh Total Score (99046), and we used both in the modeling.

**Table 2:** Model performance with different methods for constructing the target variable.

Method for Constructing the Target Variable	N (pos%)	AUC	Specificity	Sensitivity
1. F53 only	12,284 (9.9%)	0.699 ±0.011	0.930 ± 0.047	0.236 ± 0.090
2. EPDS ≥ 10 only	8809 (15.6%)	0.728 ± 0.020	0.886 ± 0.036	0.358 ± 0.094
3. PHQ9 ≥ 10 only	942 (45.1%)	0.661 ± 0.022	0.666 ± 0.071	0.541 ± 0.054
4. EPDS ≥ 10 or PHQ9 ≥ 10	8994 (18.3%)	0.739 ± 0.014	0.893 ± 0.033	0.380 ± 0.074
5. F53 or EPDS ≥ 10 or PHQ9 ≥ 10	8994 (22.1%)	0.733 ± 0.008	0.858 ± 0.030	0.446 ± 0.053
6. Same as 5, except removing patients with F32, F33, F34.1 or F53.0, or EPDS ≥ 10 or PHQ9 ≥ 10 during pregnancy	7105 (15.8%)	0.659 ± 0.019	0.890 ± 0.033	0.265 ± 0.063
7. F53 and other diagnosis code for depression (F32, F33, F34.1)	12,284 (15.9%)	0.754 ± 0.010	0.881 ± 0.024	0.450 ± 0.046

### 2.2. Constructing the Primary Data Source

We represented the raw EHR data using the National Patient-Centered Clinical Research Network (PCORnet) common data model [19], which includes tables that document patient demographics, encounters, diagnoses, laboratory results, prescriptions, and other relevant information. The PCORnet model is widely adopted across institutions, facilitating the sharing and validation of our work in other settings.

All diagnosis codes were standardized to the International Classification of Diseases (ICD-9/10), and medications were normalized using RxNORM Concept Unique Identifier (RXNORM_CUI) codes. After importing the raw data into PCORnet, we employed dbt (https://github.com/dbt-labs/dbt-core (accessed on 15 January 2025)), a general-purpose data transformation tool, to extract the predictive features and target variable. These features and the target variable were then stored in a single Parquet file, which contains data for all deliveries. Each row represents a delivery, and each column corresponds to a specific feature. When multiple measurements of the same type exist for a patient (e.g., multiple height or BMI records), we aggregated them by taking the average of each measurement type. An exception was made for EPDS/PHQ-9, for which we used the maximum score recorded to better capture the most severe presentation of symptoms. This tabular data is ideal for machine learning tasks and serves as the primary data source for the analysis presented in the remainder of the paper, hereafter referred to as the WFU dataset. Further details on creating the target variable and features are provided below.

### 2.3. Machine Learning Model

#### 2.3.1. Target Variable for Predictive Modeling

It is critical to define the target variable accurately. Previous studies have used either one or a combination of the following criteria to define PPD:

(a) Specific ICD-9/10 diagnosis codes for depression and/or anxiety [18,20,21].
(b) Screening scales, such as the Edinburgh Postnatal Depression Scale (EPDS) or Patient Health Questionnaire (PHQ) [13,15,22,23].
(c) Prescription of antidepressant medications [18,21,23].
(d) Non-pharmacological treatments including referrals to or interviews with psychologists or psychiatrists [15,18].

Previous clinical studies have highlighted nuances in using antidepressant medications as indicators for PPD [24,25]. The situation is more complicated in our hospital’s setting, as many patients seek prenatal care and delivery care within our health system but prefer to receive primary care elsewhere. As a result, using prescription data to define PPD is challenging. Similarly, obtaining complete visit data for patients across different outpatient clinics and health systems is difficult, making it unfeasible to use non-pharmacological treatment records to define PPD.

We also found it problematic to rely solely on ICD-9/10 codes. For instance, if a patient is not assigned a diagnosis in the EHR during the postpartum period, they may have sought care for depression at a different hospital or clinic, meaning they would not have an ICD code recorded in the EHR even though they were diagnosed with depression elsewhere. Conversely, if a patient had a depression diagnosis during pregnancy or before, the same diagnosis code may be carried over to the postpartum period, potentially making the code insufficiently specific for modeling purposes.

Therefore, we used screening tools, specifically the EPDS and the PHQ-9 score, as the primary method for identifying the target variable. Patients without the scores from these tools were excluded from the cohort. However, in addition to depression screening tool scores, we included the diagnosis code F53 as an identifier for the target variable (irrespective of depression screen score), since this diagnosis code is specific to postpartum depression. We systematically compared different methods for defining the target variable, and the results are presented in Section 3.1 and Table 2. For purposes of this study, an EPDS of ≥ 10 or a PHQ-9 score of ≥ 10 indicated PPD [26].

#### 2.3.2. Feature Extraction and Selection

**Figure 2:**
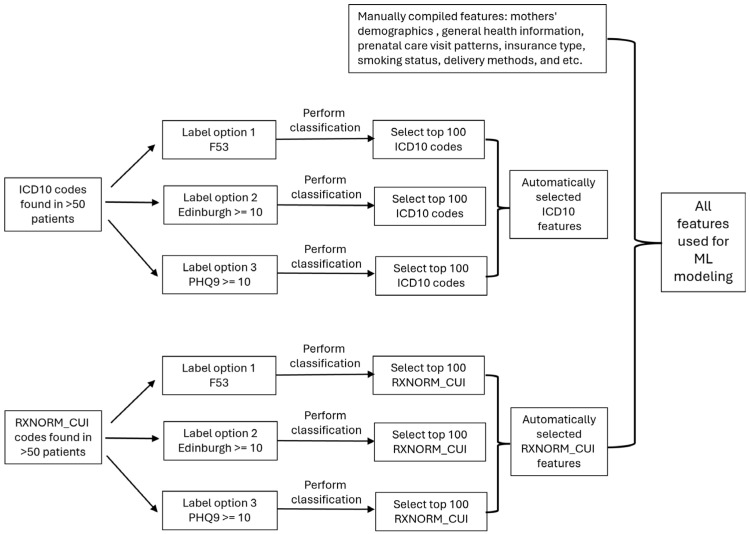
Feature Selection Workflow. The feature selection process includes: (1) Manually compiling features commonly reported in the literature; and (2) Automatically selecting ICD10 diagnosis codes and RXNORM_CUI prescription codes. For further details, see Section 2.3.2.

Figure 2 shows our workflows for feature extraction and selection. We first manually compiled a list of commonly used features from the literature, including maternal demographics and general health information, prenatal care visit patterns, insurance type, smoking status, delivery methods, and other relevant factors. We also use the following ICD-10 codes to identify patients with morbid obesity (E66.01), obesity (E66.09, E66.8, E66.9), overweight (E66.3), and obesity complicating pregnancy, childbirth, and the puerperium (O99.21). All features were calculated from 2 years prior to delivery up to the time of delivery. The statistics for these variables are presented in Table 1, which include continuous and discrete variables, respectively.

In addition to manual feature selection, we conducted an automated search for potentially useful diagnosis codes across the entire EHR dataset of the patients. Since one of our research questions involves testing various options for defining the target variables (see Section 2.3.1 and Section 3.1, and Table 2), we needed to be cautious about not introducing any bias during the automatic feature selection process. Firstly, we truncated each ICD10 code to its first digit to categorize them into broader groups and focused on those found in more than 50 patient records. We then built a random forest model (see Section 2.3.3 for details) using only the diagnosis codes as features for classification. SHAP (Shapley Additive Explanations) values were calculated for all diagnosis codes using a Python package (SHAP library version 0.46.0). We selected the top 100 codes for options 1–3 in Table 2 independently, and all selected codes were included in the final modeling process using the union of them to prevent potential bias in feature selection. Similar steps were applied to prescription records. Table S1 shows the top 10 diagnosis codes and prescriptions after our selection steps.

#### 2.3.3. Classification Modeling

In this paper, we focused on the Random Forest classification model from the Scikit-learn library. Random Forest is an ensemble learning method that constructs multiple decision trees during training. Each tree is trained on a random subset of the data, and the final prediction is made by averaging the predictions of all trees through majority voting. This model offers several advantages over other commonly used machine learning models: (a) it is consistently ranked among the top performers in the literature [13], (b) it is relatively insensitive to data normalization and feature selection methods [27,28,29], and (c) it is relatively fast to train and tune. For hyperparameter optimization, we fixed the number of trees (“n_estimators”) to 200. We used the standard 5-fold cross-validation method from the Scikit-learn library (version 1.5.1) to select the optimal “min_samples_split” value while keeping the default values for the remaining hyperparameters.

It is also worth noting that the WFU dataset is imbalanced, with 22.1% of patients being identified as positive when using method 5 to define the target variable (Table 2). To address this, we set the “class_weight” parameter to “balanced” in the Random Forest Classification model. This is similar to using class-weighted loss in boosting models, but with weights pre-calculated according to [30]. Although more sophisticated methods like focal loss [31] could be used, we believe our approach is sufficient, given that the class imbalance is not as extreme as in the examples in [31]. We did not apply any synthetic minority oversampling technique (SMOTE), as they can lead to data leakage issues if not implemented properly, as we will show later.

For model comparison, we have also evaluated performance of Logistic Regression, Support Vector Classifier (SVC) and XGBoost for the most appropriate definition for the target variable (see Section 3.1). Logistic Regression and SVC were applied after RobustScaler transformation of numerical values. Each model underwent the same evaluation steps as the Random Forest model.

#### 2.3.4. Evaluation of Model

We used the area under the curve (AUC) as the primary metric for performance evaluation. AUC is commonly employed in binary classification problems to assess the balance between the true positive rate and the false positive rate. It is also widely reported in the literature, facilitating model comparisons. Additionally, we included sensitivity and specificity scores, which are commonly used in medical literature to characterize the true positive rate and true negative rate, respectively. These metrics are also less sensitive to class imbalances compared to raw accuracy, making them more reliable indicators of model performance in the WFU dataset.

To obtain robust estimates of these metrics, we randomly split the raw dataset into training and test sets using an 80:20 ratio by rows, so no delivery contributed to both the training and test sets. We then employed stratified k-fold cross-validation (k = 5) on the training set alone, maintaining class proportions in each fold. For each run, k-1 folds were used for training and one fold for validation and hyperparameter selection. The model with the highest AUC score was selected to make predictions on the test set, where AUC, sensitivity, and specificity were calculated. Any preprocessing step (e.g., RobustScaler) and hyperparameter tuning were performed within the training set only, preventing information leakage to test set. This entire process was repeated 10 times, and each metric’s average and standard deviation were reported.

### 2.4. External Data: Census Tract Data

Both clinical and ML research suggest that the socio-economic status of patients can be an important factor in predicting PPD. For example, Ref. [7] listed socio-economic status as one of the 13 predictors in the Postpartum Depression Predictors Inventory. Ref. [16] showed that monthly income is the third most important feature in their ML model. However, less than 10% of patients have these data in our EHR. To address this gap, we incorporated census tract data from CDC (https://www.atsdr.cdc.gov (accessed on 25 March 2025)) and AHRQ websites (https://www.ahrq.gov/sdoh/data-analytics/sdoh-data.html (accessed on 25 March 2025)). In principle, census tract data provides average socio-economic information for populations living within a specific census tract or zip code. Specifically, we used the Social Vulnerability Index (SVI) from 2022 [32,33,34] and the Social Determinants of Health Database (SDOH) from 2020 [35]. Table S3 lists the features previously shown to be important and included in our model.

To integrate these data into our analysis, we first mapped the addresses provided by patients in their EHRs to the corresponding tract ID. If such a mapping was unavailable, we used the patient’s zip code instead. Only the address closest to the delivery date was considered, and if no address was provided within ±2 years of delivery, those patients were excluded from the analysis (*n* = 1048, 22%). We then looked up the relevant census tract data and added features corresponding to either the tract ID or the zip code to each patient’s records as additional columns to the WFU dataset. Models built with and without the census tract data will be compared in Section 3.3.

### 2.5. External Data: Pregnancy Risk Assessment Monitoring System (PRAMS)

One potential solution to the issue discussed in Section 2.3.1 is implementing continuous and consistent patient monitoring after delivery. While this is not feasible in a typical tertiary care hospital setting, where patients do not consistently return for follow-up visits at specific intervals within the health system for various logistical reasons, the publicly available Pregnancy Risk Assessment Monitoring System (PRAMS) dataset offers an opportunity to address this challenge. The dataset is collected by sending surveys to women who have recently delivered live births, providing valuable insights for post-delivery monitoring. We are interested in testing whether constructing an ML model based on this dataset and using it to make predictions on our dataset can improve our evaluation metrics.

The PRAMS dataset includes different phases, which span different time periods. We used Phase 7 of the dataset (2012–2015), as it was the most recent phase available at the time of our data analysis (www.cdc.gov/prams/php/data-research/index.html (accessed on 5 December 2024)). For preprocessing, we followed the steps outlined in [13], which include: (a) removing columns with more than 10,000 missing values; (b) removing any rows containing missing values after step a; (c) removing columns with only one unique value; and (d) removing highly correlated columns (correlation > 0.9). This gives a total of 41,948 patients and 192 features, which is significantly more than the WFU dataset. For the target variable, we temporarily changed our definition of PPD. We adhered to the definition in [13], which labeled patients as positive if they responded with either “always” or “often” to at least one question on the PHQ-2. This is because the only value listed in the PRAMS dataset for defining depression is PHQ-2. It is worth noting that we do not rely on PHQ-2 in our cohort (Section 2.3.1). Although PHQ-2 may be more sensitive in identifying PPD, it carries a higher risk of lower specificity—resulting in more false positives or misclassification—compared to the EPDS and PHQ-9. Section 3.4 will show whether building an ML model on the PRAMS dataset can help make robust predictions on the WFU dataset. In addition, we are interested in testing whether including a socioeconomic variable (specifically, the annual income variable INCOME7) can improve the model’s predictive performance (Section 4).

## 3. Results

### 3.1. Different Methods for Creating the Target Variables

As mentioned earlier, we systematically compared different methods for creating the target variable with the results shown in Table 2. Since we have only recently adopted the PHQ-9 as the primary screening tool in our hospital, starting in early 2023, the result based solely on PHQ-9 (method 3 in the table) is unlikely to be representative. The EPDS was used as the primary screening tool in our health system prior to 2023. Using F53 alone (method 1) may not be sensitive enough, as the positive rate of PPD is only 9.9%, far less than the estimated prevalence rate in the US (18.56%, [20]). The EPDS (method 2) or combining it with PHQ-9 (method 4) or combining EPDS, PHQ-9 and F53 (method 5) yields very similar model performance (AUC = 0.728 ± 0.020, 0.739 ± 0.014 and 0.733 ± 0.008), with the positive rate progressively increasing from method 2 to method 5.

Additionally, we tested the scenario where we only considered “new onset” PPD (method 6), as suggested by Ref. [24]. This scenario was similar to method 5, except for removing patients with ICD-10 codes F32, F33, F34.1 or F53.0, or EPDS ≥ 10 or PHQ9_total ≥ 10 during pregnancy. However, the model performance was poor, with an AUC of only 0.659 ± 0.019.

Furthermore, we also tested a method that includes all diagnosis codes for depression during the postpartum period (method 7). Although this method resulted in the highest model performance (AUC = 0.754 ± 0.010), this could be mainly due to a pre-existing diagnosis of depression being carried over into the postpartum period and provides less insight into the prediction of PPD.

For completeness, we compared our Random Forest result against different machine learning models for method 5. Logistic Regression, SVC and XGBoost yielded similar but slightly lower values (0.721 ± 0.010, 0.726 ± 0.009, and 0.725 + 0.013, respectively) over a modest grid search for hyperparameters (Table S4). These results suggest that performance differences among models are minor, with Random Forest showing slightly higher AUC. The reported value can therefore be considered representative of our feature set and target variable definition.

### 3.2. Feature Importance

From Table 1, we can see some notable, but based on prior research findings, anticipated differences between patients with and without PPD. Patients with PPD are more likely to self-report white race, smoke cigarettes, and have obesity; conversely, they are less likely to self-report Hispanic ethnicity. Patients with PPD have a lower mean maternal age and have had a higher number of healthcare visits. Patients with PPD are more likely to have been delivered by classical cesarean or have a vaginal breech delivery.

**Figure 3:**
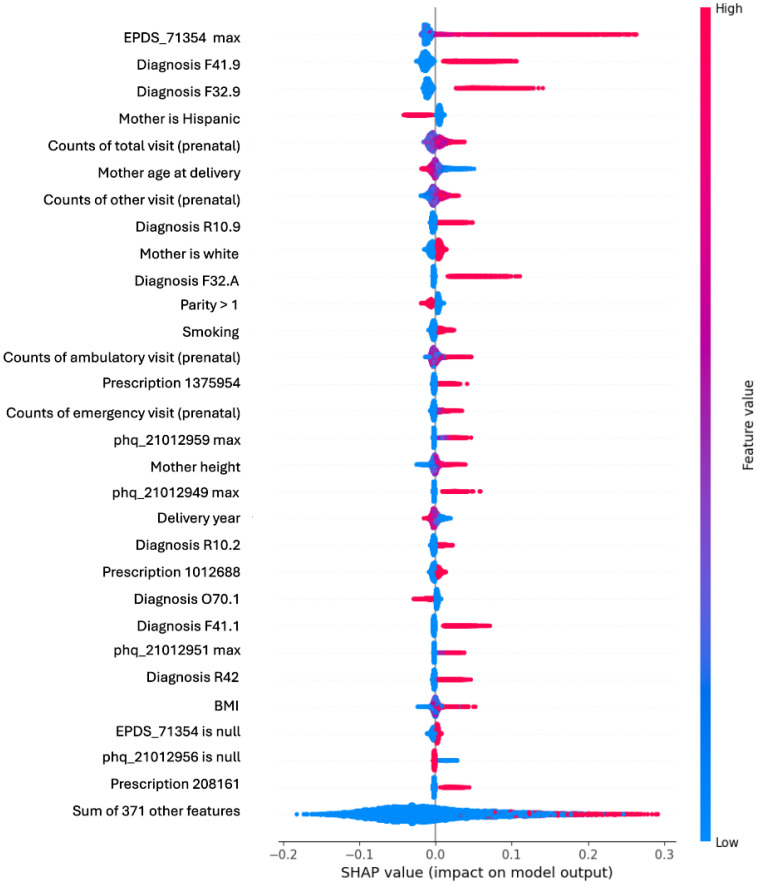
Feature importance for WFU dataset. Only the top 30 important features are shown. See Table S1 for the meanings of diagnosis and prescription codes, and Table S2 for EPDS and PHQ-9 variables.

Figure 3 presents a beeswarm plot highlighting the top 30 important features and Table S5 lists details of the SHAP values for the top 10 features. Figure S1 also shows a waterfall plot illustrating feature contributions for a randomly selected positive case. Several of these important features are expected, including pre-existing diagnoses of depression or anxiety (F41.9, F32.9, F32.A), the EPDS and PHQ9 total scores (EPDS_71354_max, phq__21012959_max), and smoking status. The number of prenatal care visits (counts_of_visits_prenatal_care) also emerged as a significant feature, suggesting that women with more complicated medical histories may have a higher likelihood of developing depression. Additionally, women experiencing pain (R10.9, R10.2) were more likely to develop PPD, although the causal relationship between pain and depression remains debated [36]. Younger women were also more prone to PPD, consistent with previous clinical studies [37]. Interestingly, Sertraline (Zoloft™, prescription_208161 in Figure 3), a medication commonly used to treat depression and with the highest unadjusted odds ratio (OR) in Table S1, ranks only 29th in terms of feature importance. This is likely due to (1) the correlation between antidepressant prescriptions and depression diagnosis codes, so the medication’s importance was ranked lower since the diagnosis codes were already used and among the top features, and (2) the fact that this category of medication is also prescribed for other pre-existing depressive disorders that are not part of the defined target variable (PPD).

In addition to antidepressants, two other medications, namely Fentanyl–Bupivacaine (2 mcg/mL–0.125% in NACL) injection and Doxylamine–Pyridoxine (10 mg each, delayed release), were also ranked among the top 30 features. These medications are not direct treatments for depression but may serve as clinical proxies or contextual markers related to PPD risk. The Fentanyl–Bupivacaine combination is commonly used for labor analgesia during epidural or spinal anesthesia, which may reflect more intervention-intensive or complicated deliveries or perhaps a patient’s variable perception of pain (i.e., pain can be perceived as more intense in the presence of depression). Previous studies have reported an association between intrapartum analgesia and subsequent mood disturbances, possibly due to pain experience, surgical recovery, or psychological responses to unexpected interventions (e.g., cesarean delivery). Doxylamine-Pyridoxine, often prescribed for nausea and vomiting of pregnancy, may be a surrogate marker for heightened symptom burden or more medically managed pregnancies. Of note, hyperemesis gravidarum, which is initially treated with Doxylamine-Pyridoxine, has been associated with depression and other mood disorders [38]. The inclusion of such medications underscores the utility of EHR-based modeling in capturing indirect clinical signals that may influence or correlate with psychological outcomes postpartum.

### 3.3. Comparison of Model Performance with and Without Census Tract Data

From Table 3, we observe that adding census tract data did not improve model performance. This finding holds whether we limited the analysis to the subset of patients with a successfully mapped tract ID (comparing data B, with an AUC = 0.714 ± 0.022, and C, with an AUC = 0.707 ± 0.019) or used the mean or zip code level to impute data for patients without a tract ID (comparing data A and D/E, with AUC = 0.725 ± 0.011, 0.721 ± 0.008, 0.719 ± 0.008, respectively).

Additionally, we conducted a direct correlation analysis between the census tract data and the target variable using the Spearman method without building any ML model. The correlation coefficients are all below 0.04, further confirming that adding census tract data will not enhance model performance.

### 3.4. Using PRAM to Build a Model to Make a Prediction on the WFU Dataset

Building a Random Forest model on the PRAMS dataset yields an AUC score of 0.730 ± 0.008 (Table 4), which is surprisingly close to the score from the WFU dataset despite many features in the PRAMS dataset not being present in ours. To identify common features for making predictions on our dataset, we first selected the top 50 important features based on the Shapley Additive Explanations (SHAP) values and reran the model with these selected features. The fact that the second run produced an almost identical AUC score (0.729 ± 0.009) to the model using all features suggests that these features are sufficient. We then manually compared these features with those in our dataset and identified the ones common to both (see Table S6 for a list of features). Rerunning the model on these common features with the PRAMS dataset resulted in an AUC score of 0.673 ± 0.004. Using the model built solely on PRAMS to make predictions on our dataset resulted in an AUC score of 0.646 ± 0.002. This demonstrates that a model built solely on PRAMS can achieve similar performance on our dataset. However, due to the lack of overlap in features between the two datasets, performance degraded significantly.

### 3.5. Model Performance Among Different Races

**Figure 4:**
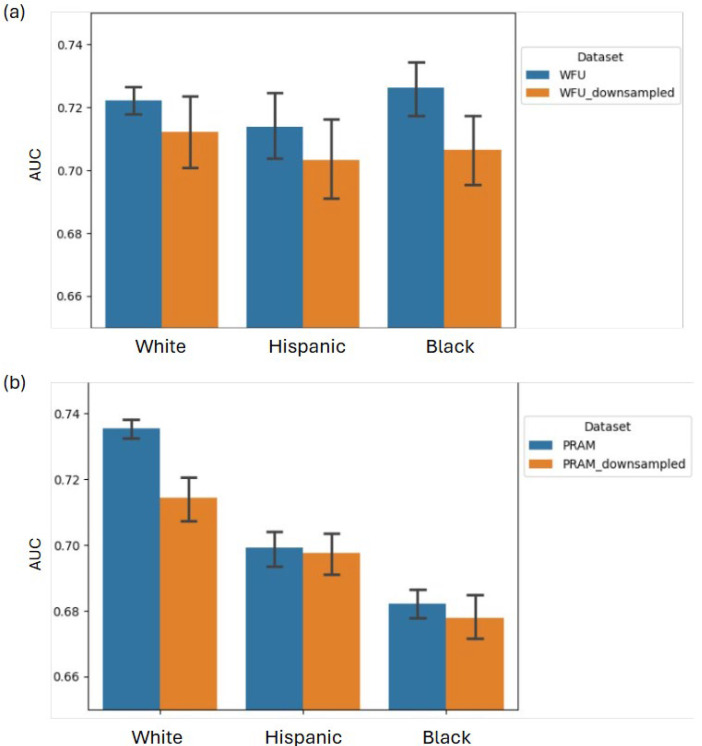
Comparison of model performance across different racial and ethnic groups for (**a**) the WFU model and (**b**) the PRAM model. Results are shown for both the raw dataset and the downsampled versions. Error bars represent the 95% confidence interval of the mean.

Similarly to the findings of [14,39], we observed differences in model performance across racial and ethnic subgroups. Given that Hispanic and Black patients comprise approximately 16% and 21% of the overall cohort, respectively, we increased the number of randomizations from 10 (see Section 2.3.4) to 50 to obtain more robust estimates of the mean and standard deviation of AUCs. Model performance across racial/ethnic groups is presented in Table 5 and Figure 4. Specifically, while performance among Black and White patients closely aligns with that of the overall cohort (AUC = 0.722 ± 0.016 and 0.726 ± 0.029, respectively), it is slightly lower among Hispanic patients (AUC = 0.713 ± 0.040), with a *p* value of 0.170 when compared to the White population. Notably, simple resampling of the dataset—reducing the number of White patients—did not fully eliminate this bias (Table 5 and Figure 4). The observation of a similar but statistically significant bias in the PRAM dataset (*p* < 0.01), likely due to its larger sample size, suggests race and ethnicity-based generalizability concerns that warrant further study. Potential causes of this bias are further discussed in Section 4.

## 4. Discussion

A comprehensive comparison of model performance with previous studies is beyond the scope of this paper. Interested readers can refer to recent reviews [12,40,41]. However, we compared our findings with recent models across EHR and survey datasets to contextualize our results.

Several studies have focused on building ML models to predict PPD within the same period as ours (1 year postpartum). For example, a UK study of 266,544 women reported an AUC score between 0.72 and 0.74 for their EHR-based model [18], closely aligning with our model’s performance. Subsequently, they incorporated the EPDS into their feature set, raising the AUC to 0.844, which is the value reported in [12] and [40]. However, since the EPDS is a screening tool for PPD and was already used in defining our target variable, including it in our feature set would be problematic.

Shin et al. [13] reported an AUC score of 0.884 using the PRAM dataset, one of the highest in the literature. However, this likely stems from the misuse of the SMOTE, which led to the calculation of model performance on an up-sampled test set rather than the original one (see Figure 1 in their paper). We confirmed this effect: applying SMOTE in the same way produced an AUC of 0.897, whereas evaluating on the original PRAMS dataset yields 0.730, consistent with the AUC of our model built on the WFU dataset.

While our model’s performance is consistent with another study [23] (AUC = 0.712), based on a cohort of 214,359 women with a new PPD onset rate of 1.9% in Israel, it consistently lags behind Zhang et al. [21], which reported an AUC of 0.886. One potential reason for this discrepancy is that Zhang et al. [21] used EHR data from a larger health system, which may have provided a more complete record of patients; however, the exact cause requires further investigation.

It is also worth pointing out that our model performs the worst when using “new onset” of PPD as the definition of the positive label (Table 2). We posit that the degraded performance is likely due to selection bias, as this method may select a lower-risk population by removing individuals with a pre-existing (pre-pregnancy or prenatal) diagnosis of depression. For instance, a patient could have a pre-existing depressive disorder and then develop postpartum depression superimposed on the chronic condition. In fact, both chronic depressive disorders and prior episodes of postpartum depression are known risk factors for developing postpartum depression. This association between chronic or prior mental health disorders and increased risk for PPD was also demonstrated in our study (Table S1). Moreover, this method decreases the generalizability of our model, as it limits the model’s applicability to patients without a history of depression.

One of the disadvantages of using method 5 to identify PPD patients (compared to method 1, see Section 2.3.1 and Section 3.1) is that we have complete data on only 73.2% (8994/12,284) of the source cohort. This evidence suggests that improved adherence to screening and recording screening results in the EHR would enhance PPD detection and potentially improve AI efforts in predicting the outcome of PPD.

One surprising finding in this study is that adding socio-economic data from census tract information did not improve model performance (Section 3.3). We can begin to explore the reasons for this using the PRAM dataset. The PRAM dataset’s INCOME7 variable (total family income in the 12 months before delivery) ranks among the top 10 important features when modeling the dataset alone and adding this variable to the model with only common features indeed improved performance to 0.682 ± 0.005 (Table 4). However, when we used census tract data to represent this variable in the WFU dataset, model performance decreased to 0.630 ± 0.002. This suggests that while socio-economic features are important predictors, using data averaged at the tract or zip code level is insufficient. Incorporating patient-level socioeconomic data, which is currently limited and often missing in the EHR, could potentially enhance model performance.

Lastly, we should note that downsampling the data to make the racial and ethnic distribution more uniform did not improve model performance among Hispanic patients (Table 5 and Figure 4). A recent study by Ref. [42] also showed that their ML model performed worse among Latina women and non-Hispanic Black women compared to non-Hispanic White women, even though non-Hispanic White women constitute only ~10% of their cohort from an urban academic hospital in Chicago. From a clinical perspective, lower detection rates of PPD in certain patient populations may reflect a combination of clinical and data capture factors. It is well-documented that PPD is often under-detected in Hispanic women due to decreased reporting and/or detection by their healthcare providers due to various personal, social, and healthcare delivery variations (see [43] for a thorough review).

We observed a similar ethnicity trend in our dataset. For example, only 83.7% of Hispanic women who delivered at our hospital had at least one prenatal care visit and one postpartum visit, and only 57.4% of them were screened using either the EPDS or PHQ-9 score. These percentages are significantly lower than those in the entire cohort, occurring 89.0% and 67.9%, respectively. The contrast in PPD positive rates (13.2% Hispanic vs. 22.1% non-Hispanic) further supports this observation.

More interestingly, when we conducted a similar subgroup analysis by insurance type, we found no difference in model performance between Medicaid and privately insured patients, both when including all patients and when restricted to those with a single insurance type (Table S7). Because insurance type often serves as a proxy for socioeconomic status, this finding suggests that the lower performance among Hispanic patients is more likely due to under-reporting or under-documentation rather than socioeconomic factors. Therefore, increased awareness of healthcare variability faced by some patients, along with enhanced screening, may provide a more effective way to identify and treat PPD for all patients.

## 5. Conclusions

In this study, we constructed an ML model using EHR data from our hospital to predict PPD. Compared to previous studies using publicly available datasets or prospective cohorts, we showed several challenges in a fragmented tertiary healthcare setting. First, we found that using EPDS and PHQ-9 scores, complemented by the specific diagnosis code F53 for PPD, is the preferred approach for creating the target variable. This method captures most positive cases, minimizes selection bias due to the absence or presence of chronic depression, and is specific enough to prevent the problem of incorrect diagnosis due to a pre-existing diagnosis being erroneously carried forward into the postpartum portion of the EHR.

Our ML model, based on Random Forest classification, achieved an AUC score of 0.733 ± 0.008, comparable to previous studies using EHR data. We also tested the addition of census tract data, which provides socio-economic statistics, and PRAMS, which offers more continuous monitoring of mothers postpartum. Although these datasets did not improve model performance, they highlighted that socio-economic data averaged at the tract or zip code level are insufficient to improve model performance, suggesting that more robust patient-level socio-economic data may be more effective in prediction. Model performance varied across subgroups, with reduced performance among Hispanic patients, which may be due to lower rates of visits and screening.

Clinically, our results suggest that machine learning-based models could complement existing screening protocols by identifying at-risk mothers who may otherwise be missed in routine care. Such tools could support earlier intervention, guide allocation of mental health resources including more frequent follow-ups, and direct initiatives to improve continuity of care in fragmented health systems. Importantly, subgroup differences highlights the need for more comprehensive data capture and consistent screening to support model reliability across various clinical scenarios [2,44].

One of our study’s current limitations is that our feature extraction relies solely on aggregated values. Future work should investigate whether incorporating a time-dependent component would be beneficial. For example, is a depression diagnosis closer to delivery more (or less) impactful or actionable than one earlier in the pregnancy, or than one in the postpartum period? Another limitation is that our study used only tabular data and missed the rich information in doctors’ and providers’ notes. Future studies can develop Natural Language Processing (NLP) tools to leverage this information. In addition, further studies are needed to understand how factors not commonly recorded in the EHR, such as hospital delivery volume [45] and travel burden [46] which have been shown to affect healthcare quality, may influence postpartum depression.

Open methodological comparisons using common data models, such as PCORnet, offer valuable insights by standardizing data and analysis methods, ensuring more consistent and reproducible research. We have intentionally built our entire data extraction and ML analysis pipeline on top of PCORnet and will open-source all the code used in this paper. This allows interested researchers to reuse and test our model with their patient data easily.

To enable clinical implementation and to support generalizability, before implementing the predictive model clinically, the model requires further external validation and prospective evaluation. We are actively collaborating with Duke University and UNC-Chapel Hill to perform cross-site validation and assess generalizability across diverse clinical populations. Following these steps, we plan to pilot the model in prenatal clinics to determine its clinical utility, provider usability, and effectiveness in guiding early interventions. These studies will inform broader implementation strategies, and feedback from real-world clinical users will be used to refine the model iteratively. This roadmap is essential for transitioning the model from retrospective research to a reliable clinical decision support tool.

We are also actively working to integrate the model into our hospital’s electronic medical record, enabling real-time risk stratification during prenatal and postpartum visits. We are also engaging clinicians to gather feedback on model outputs and usability, including through reinforcement learning approaches that incorporate provider responses to refine predictions and improve clinical relevance. Furthermore, iterative refinement based on clinician feedback in real-world settings may identify opportunities to improve model performance and contextual relevance, aligning predictions more closely with clinical needs.

## Figures and Tables

**Table 3:** Model performance when census tract data is added. (A) Baseline: the raw WFU data, but only for patients with an address within ±2 years of delivery; (B) Baseline with tract ID: baseline data with successfully mapped tract ID; (C) Baseline with tract ID + census tract: similar to Baseline with tract ID, but with census tract data listed in Table S3 added; (D) Baseline + census tract (filled with mean): similar to A, but with census tract data added. If no tract ID is found for a patient, the mean value of the corresponding census tract data for all patients was used; (E) Baseline + census tract (filled with zip): similar to D, except that when no tract ID is found, the corresponding census tract data related to the same patient’s zip code was used.

Dataset	N (pos%)	AUC	Specificity	Sensitivity
A: Baseline	7946 (22.8%)	0.725 ± 0.011	0.845 ± 0.024	0.453 ± 0.034
B: Baseline with tract ID	5153 (23.4%)	0.714 ± 0.022	0.823 ± 0.044	0.456 ± 0.046
C: Baseline with tract ID + census tract	5153 (23.4%)	0.707 ± 0.019	0.835 ± 0.052	0.430 ± 0.083
D: Baseline + census tract (filled with mean)	7946 (22.8%)	0.721 ± 0.008	0.830 ± 0.039	0.476 ± 0.061
E: Baseline + census tract (filled with zip)	7946 (22.8%)	0.719 ± 0.008	0.826 ± 0.024	0.486 ± 0.049

**Table 4:** Model performance among PRAM and WFU datasets with different selected features.

Dataset	N (pos%)	AUC	Specificity	Sensitivity
PRAM with all features	41,948 (11.0%)	0.730 ±0.008	0.836 ± 0.040	0.458 ± 0.059
PRAM with top 50 features	41,948 (11.0%)	0.729 ± 0.009	0.750 ± 0.018	0.584 ± 0.030
PRAM with common features	85,259 (12.7%)	0.673 ± 0.004	0.653 ± 0.009	0.594 ± 0.008
WFU with common features	3063 (7.4%)	0.646 ± 0.002	0.554 ± 0.011	0.643 ± 0.020
PRAM with common features + INCOME7	80,193 (12.8%)	0.682 ± 0.005	0.650 ± 0.009	0.610 ± 0.015
WFU with common features + INCOME7	2748 (7.8%)	0.630 ± 0.002	0.617 ± 0.011	0.571 ± 0.016

**Table 5:** Model performance across the PRAM and WFU datasets for different races and ethnicities. Downsampled versions of the two datasets are also used to demonstrate model performance when races/ethnicities are relatively uniform within the dataset. *p*-values are computed using two-sided *t*-test and compared to the model performance for the white patients within each dataset.

Dataset	Subset for Prediction	N (pos%)	AUC	*p* Value
WFU	Hispanic	1483 (13.2%)	0.713 ± 0.040	0.170
	Black	1960 (23.6%)	0.726 ± 0.029	0.400
	White	4600 (25.1%)	0.722 ± 0.016	reference
WFU downsampled	Hispanic	1200 (13.2%)	0.703 ± 0.047	0.275
	Black	1200 (23.6%)	0.706 ± 0.039	0.538
	White	1200 (24.5%)	0.712 ± 0.041	reference
PRAM selected race/ethnicity only	Hispanic	6539 (11.1%)	0.699 ± 0.019	<0.01
	Black	7740 (15.6%)	0.682 ± 0.016	<0.01
	White	33,307 (10.0%)	0.735 ± 0.010	reference
PRAM selected race/ethnicity only downsampled	Hispanic	6000 (11.0%)	0.697 ± 0.022	<0.01
	Black	6000 (15.5%)	0.677 ± 0.024	<0.01
	White	6000 (10.7%)	0.714 ± 0.024	reference

## Data Availability

The code used for preprocessing, modeling, and evaluation is available at https://github.com/wake-forest-ctsi/ammi (accessed on 18 September 2025). This includes scripts for cohort construction, feature extraction, and PRAMS transferability analysis.

## Supplementary Materials

The following supporting information can be downloaded at: https://www.mdpi.com/article/10.3390/jcm14186644/s1, Table S1: Same as Table 1, but for selected diagnosis codes and prescriptions. Only the top 10 most significant diagnosis codes and prescriptions are listed for clarity, along with their odds ratios (OR) and confidence intervals (CI). All variables were sorted according to their SHAP values, as described in Section 2.3.2; Table S2. Corresponding questions and meanings of variables related to PHQ and EPDS. For PHQ9, the following mapping is used to convert the answers to numerical values: 0: Not at all; 1: Several days; 2: More than half the days; 3: Nearly every day; NI: No information; Table S3. Variables and their meanings from the census tract data; Table S4. Performance comparison of Random Forest with alternative classifiers. All models underwent the same evaluation steps (Section 2.3.4) and used method 5 (Section 3.1) for target variable definition. Logistic Regression and SVC were applied after using RobustScaler transformation on numerical columns; XGBoost used raw features. Each alternate model underwent a modest grid search: Logistic Regression: C = [0.001, 0.01, 0.1, 1.0, 10, 100]; SVC: C = [0.1, 1.0, 10, 100], gamma = [‘scale’, ‘auto’]; XGBoost: n_estimators = [100, 200, 300], max_depth = [3, 5, 7], learning_rate = [0.01, 0.05, 0.1]. Reported values are mean ± standard deviation across 10 random seed realizations; Table S5. Mean SHAP values for the top 10 features and the corresponding lower and upper bound at 95% confidence intervals. The lower and upper bound were estimated using 2000 bootstrap resamples; Table S6. Variables and their meanings for the top 50 important features in the PRAMS dataset. The table also indicates whether each variable is present in the WFU dataset, as well as any special comments regarding the variable; Table S7. Model performance by insurance type. Results are shown for patients with Medicaid (payer type = 2 in PCORnet) and patients with private insurance (payer type = 51 in PCORnet). Analyses were conducted both including and excluding patients with multiple insurance designations (e.g. patients marked as both Medicaid and private, or Medicaid and self-pay); Figure S1. Waterfall plot illustrating feature contributions for a randomly selected positive case. Labels prefixed with dx_ and rx_ correspond to ICD-10 diagnosis codes and RXNORM_CUI medication codes, respectively.

## Abbreviations

The following abbreviations are used in this manuscript:

PPD	Postpartum depression
ML	Machine Learning
EHR	Electronic Health Record
AUC	Area Under the Receiver Operating Characteristic Curve
PRAMS	Pregnancy Risk Assessment Monitoring System
PCORnet	Patient-Centered Clinical Research Network
ICD	International Classification of Diseases
EPDS	Edinburgh Postnatal Depression Scale
PHQ	Patient Health Questionnaire
SHAP	Shapley Additive Explanations
WFU	Wake Forest University
SVI	Social Vulnerability Index
SDOH	Social Determinants of Health Database
OR	Unadjusted Odds Ratio
SMOTE	Synthetic Minority Over-sampling Technique
