# Supplementary material for: Building a Machine Learning Model to Predict Postpartum Depression from Electronic Health Records in a Tertiary Care Setting
Source: J Clin Med. 2025 Sep 20;14(18):6644. doi: 10.3390/jcm14186644 (PMC12470393; doi:10.3390/jcm14186644)

Supplementary Materials for "Building a Machine Learning Model to Predict Postpartum Depression from Electronic Health Records in a Tertiary Care Setting"

Table S1. Same as Table 1, but for selected diagnosis codes and prescriptions. Only the top 10 most significant diagnosis codes and prescriptions are listed for clarity, along with their odds ratios (OR) and confidence intervals (CI). All variables were sorted according to their SHAP values, as described in Section 2.3.2.

Variable	PPD (N=1986; 22.1%)	Non-PPD (N=7008; 77.9%)	OR(CI)
Diagnosis			
F32.9: Major Depressive Disorder	531 (26.7)	630 (9.0)	3.69 (3.25,4.20)
F32.A: Depression, unspecified	226 (11.4)	271 (3.9)	3.19 (2.66,3.84)
F41.1: Generalized Anxiety Disorder	182 (9.2)	239 (3.4)	2.86 (2.34,3.49)
F41.9: Anxiety Disorder, Unspecified	687 (34.6)	1100 (15.7)	2.84 (2.54,3.18)
M54.5: Low back pain	261 (13.1)	566 (8.1)	1.72 (1.47,2.01)
O70.1: Second degree perineal laceration during delivery	251 (12.6)	1176 (16.8)	0.72 (0.62,0.83)
O80: Encounter for full-term uncomplicated delivery	929 (46.8)	3552 (50.7)	0.86 (0.77,0.94)
R10.2: Pelvic and perineal pain	501 (25.2)	1240 (17.7)	1.57 (1.39,1.77)
R10.9: Unspecified abdominal pain	509 (25.6)	1131 (16.1)	1.79 (1.59,2.02)
R42: Dizziness and giddiness	276 (13.9)	559 (8.0)	1.86 (1.60,2.17)
Prescription			
1012688: Fentanyl (PF) 2 mcg/ml-Bupivacaine 0.125 %-nacl injection solution	908 (45.7)	2852 (40.7)	1.23 (1.11,1.36)
104098: Misoprostol 200 mcg tablet	300 (15.1)	1296 (18.5)	0.78 (0.68,0.90)
104895: Ondansetron hcl 4 mg tablet	529 (26.6)	1297 (18.5)	1.60 (1.42,1.80)
1375954: doxylamine 10 mg-pyridoxine (vit b6) 10 mg tablet,delayed release	465 (23.4)	1150 (16.4)	1.56 (1.38,1.76)
1483571: docusate sodium 100 mg capsule	335 (16.9)	848 (12.1)	1.47 (1.28,1.69)
208161: sertraline 50 mg tablet	190 (9.6)	231 (3.3)	3.10 (2.54,3.79)
351638: butalbital-acetaminophen-caffeine 50 mg-325 mg-40 mg tablet	200 (10.1)	357 (5.1)	2.09 (1.74,2.50)
543951: lidocaine hcl 10 mg/ml (1 %) injection solution	438 (22.1)	1733 (24.7)	0.86 (0.76,0.97)
828350: cyclobenzaprine 10 mg tablet	387 (19.5)	817 (11.7)	1.83 (1.61,2.09)
886624: butorphanol 2 mg/ml injection solution	717 (36.1)	2281 (32.5)	1.17 (1.05,1.30)

Table S2. Corresponding questions and meanings of variables related to PHQ and EPDS. For PHQ9, the following mapping is used to convert the answers to numerical values: 0: Not at all; 1: Several days; 2: More than half the days; 3: Nearly every day; NI: No information.

Variable name	Meaning
phq_21012948	Little interest or pleasure in doing things in last 2 weeks
phq_21012949	Feeling down, depressed, or hopeless in last 2 weeks
phq_21012950	Trouble falling or staying asleep, or sleeping too much in last 2 weeks
phq_21012951	Feeling tired or having little energy in last 2 weeks
phq_21012953	Poor appetite or overeating in last 2 weeks
phq_21012954	Feeling bad about yourself - or that you are a failure or have let yourself or your family down in last 2 weeks
phq_21012955	Trouble concentrating on things, such as reading the newspaper or watching television in last 2 weeks
phq_21012956	Moving or speaking so slowly that other people could have noticed. Or the opposite - being so fidgety or restless that you have been moving around a lot more than usual in last 2 weeks
phq_21012958	Thoughts that you would be better off dead, or of hurting yourself in some way in last 2 weeks
phq_21012959	PHQ9/PHQ9A Total Score
EPDS_71354	Edinburgh Postnatal Depression Scale Total
EPDS_99046	Edinburgh Total Score

Table S3. Variables and their meanings from the census tract data.

Variable	Meaning
epl_pov150	Percentile percentage of persons below 150% poverty estimate
epl_unemp	Percentile percentage of civilian (age 16+) unemployed estimate
epl_hburd	Percentile percentage of housing cost-burdened occupied housing units estimate
epl_nohsdp	Percentile percentage of persons with no high school diploma (age 25+) estimate
epl_uninsur	Percentile percentage of uninsured estimate
rpl_theme1	Percentile ranking for Socioeconomic Status theme summary
rpl_theme2	Percentile ranking for Household Characteristics theme summary
rpl_theme3	Percentile ranking for Racial and Ethnic Minority Status theme
rpl_theme4	Percentile ranking for Housing Type/ Transportation theme
rpl_themes	Overall percentile ranking
acs_median_hh_inc	Median household income (dollars, inflation-adjusted to data file year)
acs_pct_inc50	Percentage of population with income to poverty ratio under 0.50
acs_pct_person_inc_100_124	Percentage of population with an income to poverty ratio between 1.00 and 1.24
acs_pct_person_inc_125_199	Percentage of population with an income to poverty ratio between 1.25 and 1.99
acs_pct_person_inc_above200	Percentage of population with an income to poverty ratio of 2.00 or higher
acs_pct_person_inc_below99	Percentage of population with an income to poverty ratio under 1.00
acs_pct_college_associate_dgr	Percentage of population with some college or associate's degree (ages 25 and over)
acs_pct_bachelor_dgr	Percentage of population with a bachelor's degree (ages 25 and over)
acs_pct_no_work_no_schl_16_19	Percentage of teens and adults who are unemployed and not in school (between ages 16 and 19)
acs_pct_graduate_dgr	Percentage of population with a master's or professional school degree or doctorate (ages 25 and over)
acs_pct_hs_graduate	Percentage of population with only high school diploma (ages 25 and over)
acs_pct_lt_hs	Percentage of population with less than high school education (ages 25 and over)
acs_pct_posths_ed	Percentage of population with any postsecondary education (ages 25 and over)

Table S4. Performance comparison of Random Forest with alternative classifiers. All models underwent the same evaluation steps (Section 2.3.4) and used method 5 (Section 3.1) for target variable definition. Logistic Regression and SVC were applied after using RobustScaler transformation on numerical columns; XGBoost used raw features. Each alternate model underwent a modest grid search: Logistic Regression: C = [0.001, 0.01, 0.1, 1.0, 10, 100]; SVC: C = [0.1, 1.0, 10, 100], gamma = ['scale', 'auto']; XGBoost: n_estimators = [100, 200, 300], max_depth = [3, 5, 7], learning_rate = [0.01, 0.05, 0.1]. Reported values are mean ± standard deviation across 10 random seed realizations.

**Model**	**AUC**	**Specificity**	**Sensitivity**
Random Forest	0.733±0.008	0.858±0.030	0.446±0.053
Logistic Regression	0.721±0.010	0.737±0.008	0.588±0.020
SVC	0.726±0.009	0.781±0.009	0.557±0.015
XGBoost	0.725+0.013	0.773±0.011	0.565±0.019

Table S5. Mean SHAP values for the top 10 features and the corresponding lower and upper bound at 95% confidence intervals. The lower and upper bound were estimated using 2000 bootstrap resamples.

Feature	Mean Value	Lower Bound	Upper Bound
EPDS_71354 max	0.0200	0.0193	0.0207
Diagnosis F41.9	0.0194	0.0191	0.0197
Diagnosis F32.9	0.0172	0.0167	0.0175
Mother is Hispanic	0.0085	0.0084	0.0087
Counts of total visit (prenatal)	0.0061	0.0060	0.0062
Mother age at delivery	0.0056	0.0054	0.0057
Counts of other visit (prenatal)	0.0055	0.0054	0.0056
Diagnosis R10.9	0.0053	0.0052	0.0054
Mother is white	0.0052	0.0052	0.0053
Diagnosis F32.A	0.0050	0.0049	0.0052

Table S6. Variables and their meanings for the top 50 important features in the PRAMS dataset. The table also indicates whether each variable is present in the WFU dataset, as well as any special comments regarding the variable.

Variable	Definition from PRAMS codebook	Found in WFU dataset	Comment
BPG_DEPRS	before pregnancy health prob -- depression	Yes	
STRS_ARG	stress -- argue lots		
STRS_TT3	var: total number of stresses		
STRS_T_G	var: total number stresses grouped		
MAT_DEG	maternal highest degree		
PRE_DEPR	pre-pregnancy check for depression/anxiety		
INCOME7	income -- 12 mnths bef total income	Yes	use census tract data
BF5WEEKS	var: number of weeks breastfed baby		
STRS_BIL	stress -- couldnt pay bill		
MAT_AGE_PU	maternal age grouped	Yes	
WIC_PREG	wic -- dur preg		
WTTWO	nonresponse wt		definition unclear
MAT_ED	maternal education		
PP_MEDIC	insurance paid by -- medicaid (forced skip)		
MARRIED	marital Status		
PAY	method of payment	Yes	
INSMED	insurance paid by -- medicaid	Yes	before pregnancy
INSWORK	insurance paid by -- job	Yes	before pregnancy
HI_WORK	insurance paid by -- job	Yes	post-partum period
HI_MEDIC	insurance paid by -- medicaid	Yes	post-partum period
PGWT_GN	preg weight gain/lost	Yes	
MAT_WIC	mother get wic food during pregnancy?		
PREG_TRY	preg -- trying		
STRS_DRG	stress -- others drugs		
DDS_PROB	dds -- needed to see for problem		
PP_WORK	insurance paid by -- job (forced skip)		
TOTCNT	bc wt: total count		definition unclear
MOM_BMI	var: mom body mass index from qx	Yes	
SAMCNT	bc wt: sample count		definition unclear
SMK6C_NW	var: change smoking last 3 month & now	Yes	
WTONE	bc wt: sample weight		definition unclear
SUD_NEST	sudaan nest variable		definition unclear
PRE_RX	pre-pregnancy prescrip meds	Yes	
PNC_1ST	1st pnc visit -- number		

PGINTENT	preg -- intention		
SMK2YRS	smk -- >=1 cigs last 2 yrs	Yes	
PRE_DIET	pre-pregnancy dieting		
STRS_MOV	stress -- moved		
STRS_PG	stress -- husb/part preg no		
SMK6NW_A	var: yes/no smoke now	Yes	
WTANAL	bc wt: analysis wt		definition unclear
MOMLBS	maternal weight gain (lbs)		repeat PGWT_GN
MOM_BMIG_BC	var: mom bmi grouped from bc		
MAT_PRWT	maternal weight - prepregnancy	Yes	
FRACE_WHT	paternal race - white		
INFQ_AGE_MOD	var: inf age (days) quest completed (cleaned)		
MOM_WT	var: mom wt before pregnancy		repeat MOM_PRWT
PNC_WKS	var: weeks 1st pnc visit	Yes	
VITAMIN	multivitamin -- number of times /wk		

Table S7. Model performance by insurance type. Results are shown for patients with Medicaid (payer type = 2 in PCORnet) and patients with private insurance (payer type = 51 in PCORnet). Analyses were conducted both including and excluding patients with multiple insurance designations (e.g. patients marked as both Medicaid and private, or Medicaid and self-pay).

Subgroup	N (pos%)	AUC	p value
Medicaid	4311 (22.5%)	0.731±0.016	reference
Private Insurance	4702 (22.9%)	0.727±0.020	0.23
Medicaid Only	2393 (23.4%)	0.718±0.023	reference
Private Insurance Only	878 (24.2%)	0.723±0.043	0.45

Figure S1. Waterfall plot illustrating feature contributions for a randomly selected positive case. Labels prefixed with dx_ and rx_ correspond to ICD-10 diagnosis codes and RXNORM_CUI medication codes, respectively.

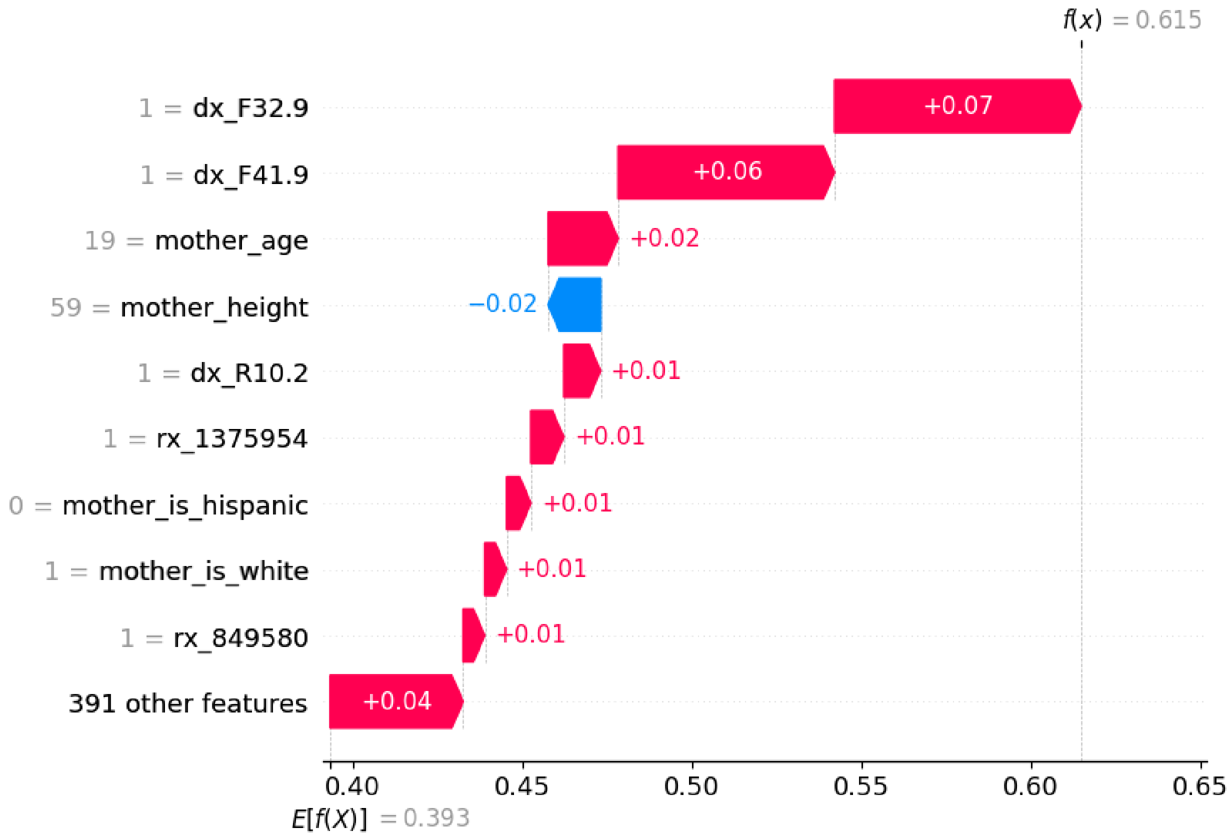

Supplement: Supplementary file 1 [file jcm-14-06644-s001.zip › jcm-3796844-supplementary.pdf]
